# A Wrist Sensor Sleep Posture Monitoring System: An Automatic Labeling Approach

**DOI:** 10.3390/s21010258

**Published:** 2021-01-02

**Authors:** Po-Yuan Jeng, Li-Chun Wang, Chaur-Jong Hu, Dean Wu

**Affiliations:** 1 Department of Electrical and Computing Engineering, National Chiao Tung University, Hsinchu 30010, Taiwan; lses40311@gmail.com; 2 Department of Neurology, School of Medicine, College of Medicine, Taipei Medical University, Taipei 11031, Taiwan; chaurjongh@tmu.edu.tw (C.-J.H.); tingyu02139@gmail.com (D.W.); 3 Department of Neurology, Shuang-Ho Hospital, Taipei Medical University, New Taipei City 23561, Taiwan; 4 Taipei Neuroscience Institute, Taipei Medical University, New Taipei City 23561, Taiwan; 5 Sleep Center, Shuang-Ho Hospital, Taipei Medical University, New Taipei City 23561, Taiwan

**Keywords:** IoT, wearable device, machine learning, streaming data, sleep posture

## Abstract

In the hospital, a sleep postures monitoring system is usually adopted to transform sensing signals into sleep behaviors. However, a home-care sleep posture monitoring system needs to be user friendly. In this paper, we present iSleePost—a user-friendly home-care intelligent sleep posture monitoring system. We address the labor-intensive labeling issue of traditional machine learning approaches in the training phase. Our proposed mobile health (mHealth) system leverages the communications and computation capabilities of mobile phones for provisioning a continuous sleep posture monitoring service. Our experiments show that iSleePost can achieve up to 85 percent accuracy in recognizing sleep postures. More importantly, iSleePost demonstrates that an easy-to-wear wrist sensor can accurately quantify sleep postures after our designed training phase. It is our hope that the design concept of iSleePost can shed some lights on quantifying human sleep postures in the future.

## 1. Introduction

Sleep is one of the most important daily activities. A night of poor quality sleep can make a person feel fatigue on the next day. Long-term sleep disorders will even induce a range of health problems [1]. It has been reported that sleep duration is a risk factor for cardiovascular disease [2]. Recent studies also reveal the relation between poor sleep quality and diabetes [3], hypertension [4] and depression [5]. Besides sleep duration, sleep habits are another indicator of sleep quality [6,7]. Sleep postures are a habit that can cause some health problems. For example, obstructive sleep apnea (OSA) can cause breath pauses during sleep. Various research works have been reported to prevent OSA through complicated system design [8,9]. In fact, this can be achieved by a simple sleep posture monitoring method [10].

**Figure 1:**
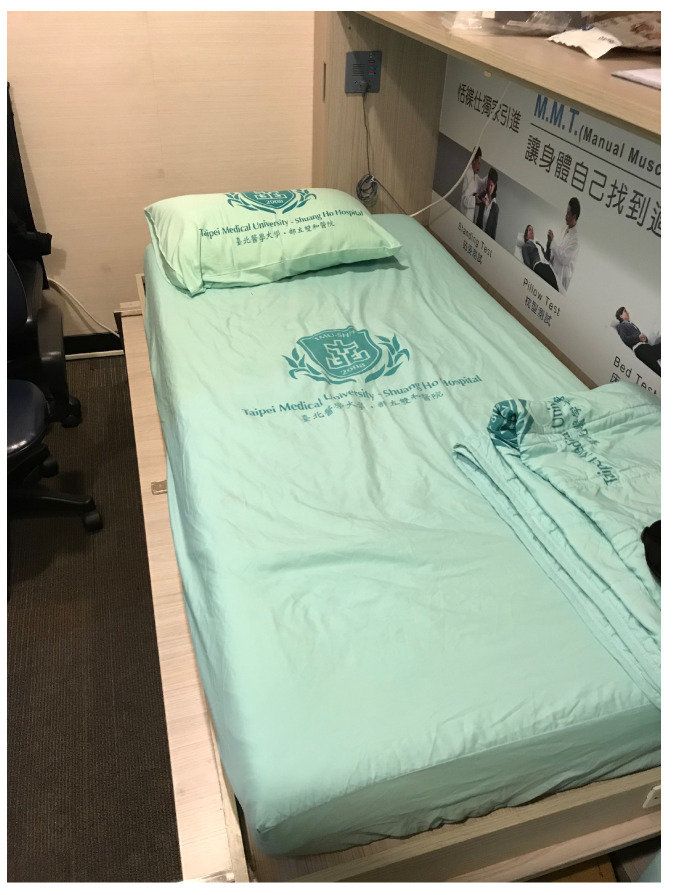
Sleep monitoring room in the sleep center. The patients suffer from sleep disease are closely monitored in this room during sleep.

Hence, sleep research centers have become an important institution in hospitals. Taipei Medical University, Shuang Ho Hospital established the first Sleep Research Center in Taiwan. Unlike patients of other traditional diseases, the patients of this center sleep overnight in a sleep monitoring room (as shown in Figure 1). During overnight sleep, multiple sensors are attached to the patient to collect biological data, including electroencephalography (EEG), electrocardiography (ECG), blood pressure, sleep postures, and so on.

**Figure 2:**
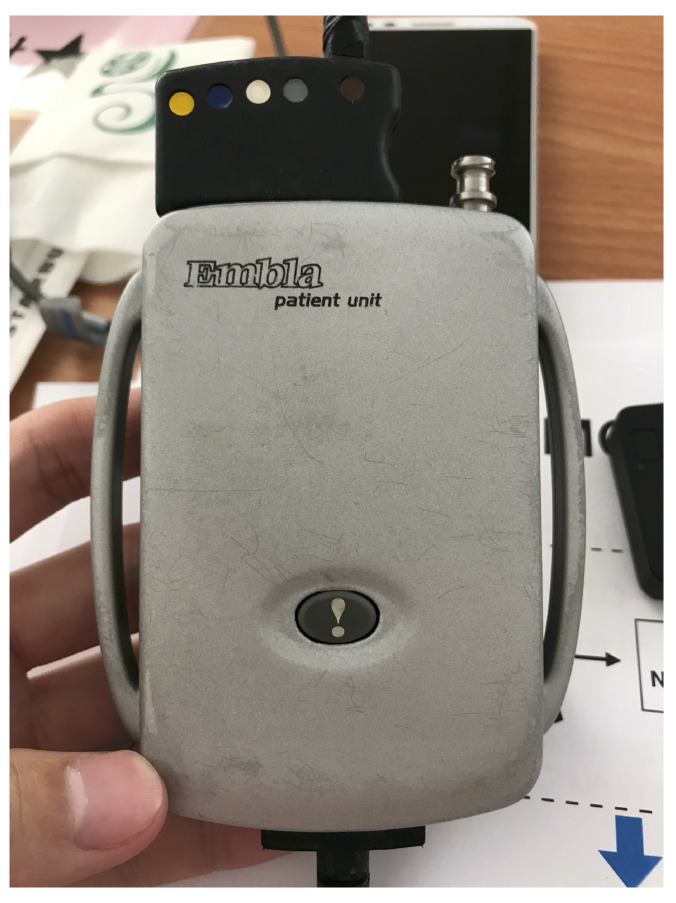
The attached device on the patient’s chest for monitoring body movement. The dimension of the device makes it a major disturbance during sleep.

Most current sleep monitoring devices are complex and inconvenient. They also disturb the patient’s sleep quality. One important monitoring device of a current sleep monitoring system is actually attached to the patient’s chest as shown in Figure 2. This chest device has an inertial measurement sensor for monitoring patients’ movements. The sensed data from this device are recorded and further analyzed to estimate sleep postures and sleep quality. This chest-worn device needs to be attached to the patient’s chest, resulting in considerable sleep disturbance. Moreover, a patient definitely feels more comfortable to sleep in his/her own home than in the center of a hospital. As a result, a more comfortable monitoring device is a desirable feature for the sleep disorder treatment business.

To this end, we have developed a novel wrist-worn sleep posture monitoring system. Current commercial wristband products, such as MI Band, Garmin Smartwatch and Apple Watch, can not recognize sleep postures [11,12,13]. Although some research studies are focusing on sleep posture monitoring, their data are generated from labor-intensive labeling, which is not quite practical [14,15].

**Figure 3:**
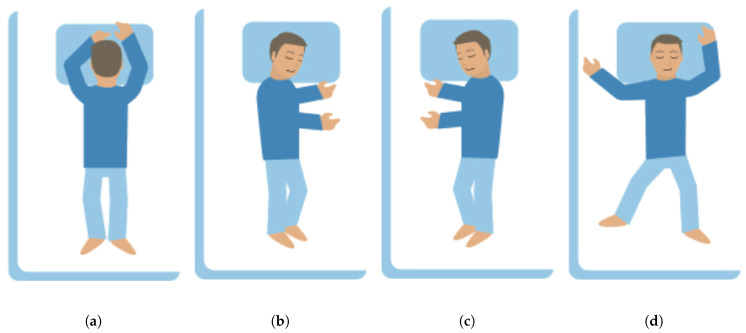
Four common sleep postures for iSleepPost to recognize. (**a**) Prone, (**b**) left lateral, (**c**) right lateral and (**d**) supine [18].

In this paper, we propose a wrist-worn sleep posture quantification system, called iSleePost, which is based on our pilot study and preliminary experiment [16,17]. During the training phase, iSleePost uses two accelerometers. The care recipient wears one accelerometer on his/her chest for automatic label collection. The other one is worn on the wrist for collecting motion data. Based on a sliding window approach, iSleePost processes the wrist-sensor data to obtain the features of a body’s motions. Based on the chest accelerometer sensor, the features are mapped to the current body position. We built the iSleePost testbed with physical accelerometers, phone and server to test the feasibility of the proposed idea. Figure 3 shows four sleep postures: supine, stomach, right side and left side [18]. From our experimental results, iSleePost can recognize and continuously monitor such four sleep postures. Two machine learning algorithms, Support Vector Machine and Random Forest, are applied for sleep posture prediction in our experiments. Our results show that iSleePost can predict sleep posture with the overall accuracy up to 85 percent.

The rest of this paper is organized as follows. The related works are discussed in Section 2. The system model is described in Section 3. Our data processing framework is presented in Section 4. The learning algorithms for iSleepPost system are discussed in Section 5. Numerical results on collected data are reported in Section 6. The future applications are discussed in Section 7. Finally, we give our concluding remarks in Section 8.

## 2. Related Works

In this section, we give a brief survey of the existing sleep posture monitoring research, which can be categorized into two kinds: contact-body class and non-contact body class. In the contact-body class, the monitoring equipment is deployed at the care recipient’ body. On the other hand, the non-contact sleep posture monitoring system does not contact the recipient’s body. Now we briefly introduce the monitoring equipment used in each work. Table 1 summarizes and briefly introduces the related works.

### 2.1. Non-Contact Body Class

For the non-contact sleep posture monitoring systems, the comfort level of the care recipient is enhanced during sleep. However, this kind of sleep postures monitoring system may face the difficulty of reducing the effects with some body-irrelevant objects, such as blankets or clothes. In addition, the recognition result of non-contact body devices will be affected by the position and angle of the monitoring equipment. Therefore, such systems are usually not portable and require sophisticated deployment processes.

Most of the research in this category adopts a surveillance camera to monitor care recipients. A video camera or image recording device is set up to continuously record images of care recipients. An image taken from the camera is processed in real-time using an image recognition-based algorithm to obtain a sleep posture [19]. Besides, vision-based approaches, depth cameras were adopted for monitoring sleep postures. In Ref. [20,21], depth cameras were used to capture the a 3D image of sleep. By analyzing the depth data, the system can recognize multiple sleep postures.

Research effort through non-camera-based equipment was proposed to monitor human activities. Wi-Fi is one of the most popular signals for activity monitoring [29]. A pair of Wi-Fi transceivers is deployed to capture the channel state information and the respiration and movements of sleeping subjects are monitored [22]. Deploying such a system requires considerable efforts. The position and angle of the equipment need to follow strict instructions to avoid interference. In addition, due to changes in the environment, a complete model training process is required after deployment. As a result, the aforementioned difficulties make them almost impossible to use at home.

### 2.2. Contact Body Class

The contact body class is a more direct approach to monitor sleep postures. Thus, most current sleep posture monitoring systems belong to this category. To clearly introduce these works, we further categorize them into two sub-categories: non-wearable and wearable. On one hand, a non-wearable subclass, such as a pressure mattress type sleep posture monitoring system, is characterized in that the care recipient does not have to wear any equipment. On the other hand, the wearable sub-class is characterized by the fact that the care recipients need to wear a monitoring device.

#### 2.2.1. Non-Wearable Sub-Class

The non-wearable sub-class of sleep posture monitoring usually adopted pressure mats as the equipment. A pressure mat is a mattress or mattress topping which has dozens of pressure sensors embedded beneath so that the distribution of the subject’s weight on the mat can be measured. A lot of research adopted pressure mats as their primary monitoring equipment for sleep posture recognition. In Ref. [23], the authors extracted 55 types of features from the raw data, which are collected from pressure mat, and then applied four classification algorithms for sleep posture estimation. In Ref. [24], the authors developed a sleep posture recognition algorithm by using limbs’ characteristics. Furthermore, [25] adopted hydraulic sensors to substitute pressure sensors for improving the convenience of the sleep monitoring system. Ref. [26] adopted a single piezoelectric sensor to measure the ballistocardiogram signals, and further to estimate sleep postures. Last but not least, [27] combined pressure mat and infrared array sensors to obtain sleep postures.

#### 2.2.2. Wearable Sub-Class

In this sub-class, care recipients need to wear a monitoring device on their bodies. Obviously, this approach can provide more accurate results than other methods. However, one of the disadvantages of this method is that wearing the device can affect the quality of sleep of the subject. In Ref. [14], the authors placed an accelerometer on the chest of the care recipient for inertial data collection. In the sleep of a care recipient, the camera recorded his/her sleep postures. Then, a linear discriminant algorithm was adopted for training a posture prediction model. the trained model was then deployed in real-time posture monitoring during sleep. A product for sleep posture monitoring with chest sensor device is actually on the market now [28]. This product exploits a disposable sensor that was worn on the chest of the care recipient.

In general, the wearable sub-class is irritating during sleep because their activities may be limited by the worn sensors, and causing further problems. However, the changing the location of the sensors could significantly reduce the inconvenience. In Ref. [15], the subject wore a tilt sensor on the wrist for recording the wrist position. Apparently, wearing a sensor on his/her wrist is more comfortable than wearing it on the chest. The experiment lasted ten nights, and then the collected data were analyzed. The result shows that a relationship exists between the sensor tilt and the body postures.

Despite the increasing popularity of sleep research, it is rarely seen that a healthy sleep posture monitoring system can simultaneously achieve the goals of high accuracy, low deployment cost, and high sleep quality. More importantly, most current methods require a labor-intensive data collection process. In this paper, we propose an innovative sleep posture monitoring system based on a wrist-worn device, which has the advantages of low deployment cost, high precision, and user-friendly sensor placement design.

## 3. System Architecture

**Figure 4:**
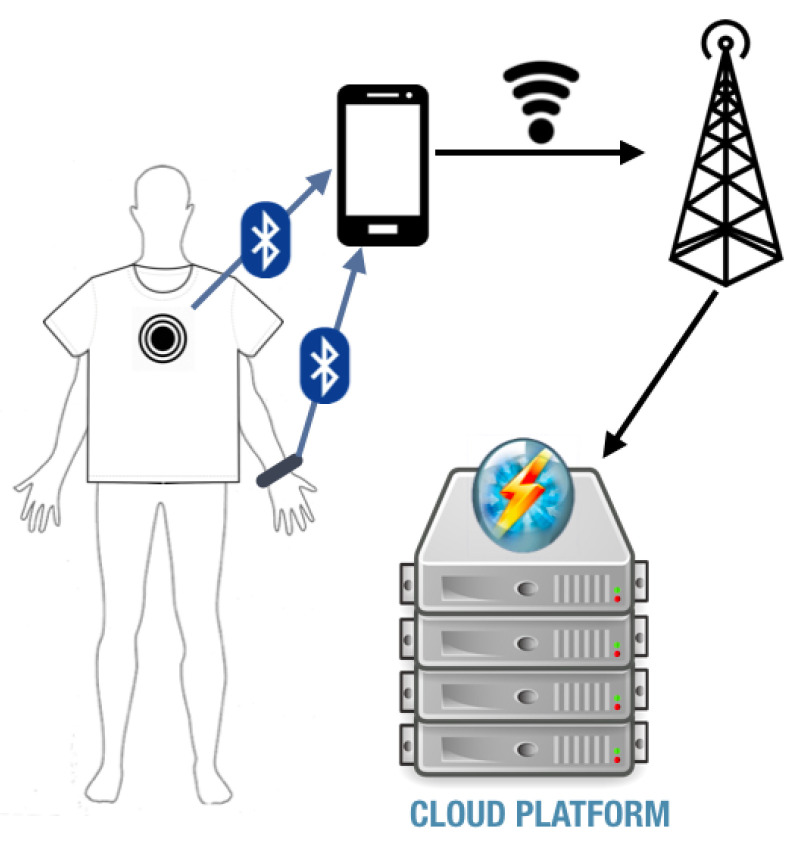
The system architecture of iSleePost, consisting of two wearable accelerometer sensors with BLE chip, a smartphone, and a personal computer connected to the Internet.

Figure 4 shows the architecture of our considered sleep posture monitoring system. Now we describe the hardware components and the communication methods between these components.

### 3.1. Hardware

The hardware of our proposed system can be divided into three layers: sensing layer, data collecting layer, and cloud layer. Devices with different computing capabilities are adopted to meet the needs of each of the layers.

#### 3.1.1. Sensing Layer

In the sensing layer, two inertial sensors are applied for sensing the motions of different body parts. The se sensors should be small and light enough to reduce the disturbance. They should be energy efficient, thereby reducing the frequency of battery replacement. In order to meet these requirements, we adopt Koala as the sensing device for this layer [30]. Koala is an inertial sensor that has been successfully applied for body motion monitoring, such as gait detection [31]. Koala is capable to detect and record physical motions, ranged from −2 G to 2 G in X, Y and Z directions. Moreover, a Bluetooth low energy (BLE) chip is attached to Koala to transmit the data with low energy consumption. The specification of Koala reports that their sampling rate is tunable, which can be set to a low frequency for long operation time. In our system, the sampling rate was set to 60 Hz.

#### 3.1.2. Data Collecting Layer

The role of the data collection layer is to collect and preprocess the raw data from the sensing layer and then send them to the designated server through the Internet. This layer plays an important role in our system. It transforms the amount of raw data into a smaller feature data, which relieves the communications load on the Internet. The device of this layer should be portable to be used at home. Hence, smartphones are the perfect candidate for the data collection layer.

#### 3.1.3. Cloud Layer

On the other end of the Internet, we need to deploy a powerful device to train models and predict sleep postures for a group of the care recipient. We used a personal computer equipped with an Intel i7 processor as the server in the cloud layer in our experimental system.

### 3.2. Communications

Most of the devices in our system are wirelessly connected for making the system portable. Depending on the capability of devices, different communication methods are considered. To reduce the energy consumption of data transmission between the sensing layer and the data collecting layer, Bluetooth Low Energy (BLE) protocol is adopted and implemented in our system. BLE is a newly announced standard for reducing energy consumption in data exchange between Bluetooth devices. As for the communication between the data collecting layer and the cloud layer, the transmitted data can be sent to the Internet via either Wi-Fi or a cellular network from smartphones. Hence, the developed sleep monitoring system can be operated in many environments.

In conclusion, the hardware components of the proposed iSleepPost system include two wearable acceleration sensing devices with Bluetooth communication function, a smart phone, and a personal computer connected to the Internet.

## 4. Data Processing Framework

**Figure 5:**
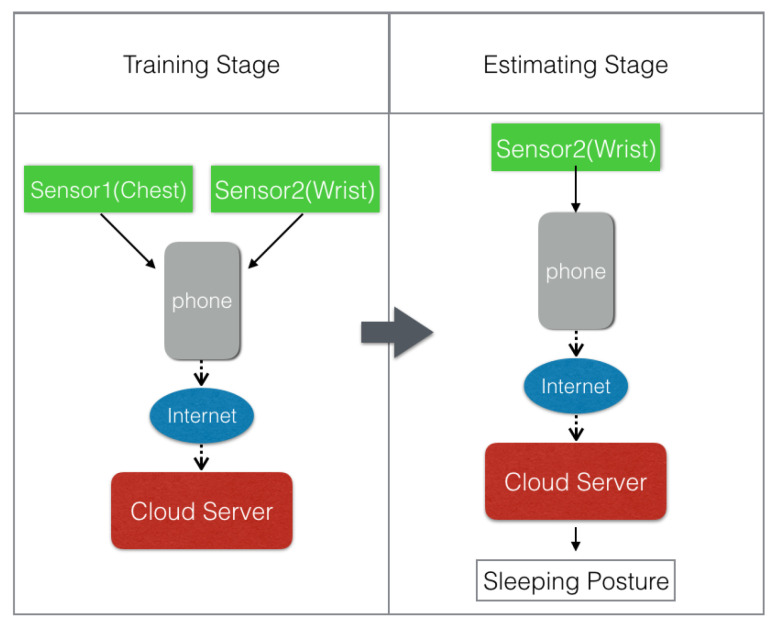
The two operation phases for iSleePost monitoring system: (1) training phase, (2) monitoring phase. In the training phase, both chest and wrist sensors are required. In the monitoring phase, only wrist sensor is required.

We designed a two-phase framework for processing the collected data. The first phase is a training phase. In this phase, one sensor is placed on the chest and the other sensor is strapped on the wrist. After training the model, the monitoring phase is initiated, and only the wrist sensor is needed. The changes in architecture between two phases are shown in Figure 5.

**Figure 6:**
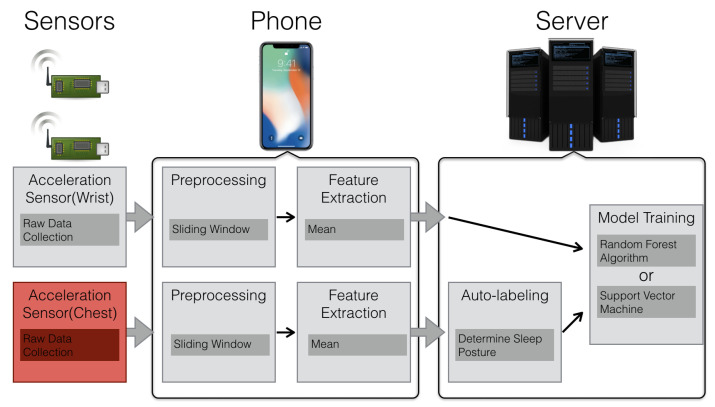
Overview of the data processing framework. The flow chart represents the pipelines of processing data. The device icons above the flow chart show the devices in which the processing elements takes place.

We have designed data pipelines to process the raw sensor data in a timely fashion. We will introduce the data pipelines and the deployment of elements in this section. The overview of data processing framework is shown in Figure 6. The flow chart in the figure shows the processing pipelines. The boxes represent the processing elements and their goals. Above the flow chart are the devices in which the elements are implemented. The processing elements are distributed across the system to reduce network traffic. The arrows indicate the directions of data flows in the framework. At the beginning of the flow are two data collecting sensors. The collected data from the wrist and chest sensors are then processed in two different ways and presented by the upper half and the lower half in the flow chart.

The chest sensor data flow is only activated when the system is in the training phase. During the training phase, the sensor data from two accelerometers are being collected simultaneously. The n the data are processed for posture model training. The wrist sensor data are processed to wrist position features, and the chest sensor data are processed for estimating the actual posture. In the monitoring phase, we estimate the sleep postures by the features of the wrist sensor data and the learned posture model. In this case, the data flow from the chest sensor will not be used.

In the next section, we will present the processing methods for the chest and wrist sensors data, respectively.

### 4.1. Chest Sensor

The orientation of a tightly tied chest sensor can represent the body position, which is the standard method for obtaining sleep posture in hospitals nowadays. Hence, the chest sensor data are used as the ground truth to train and assess the system. In addition, the chest sensor has to be securely attached to the chest the entire time; otherwise, the result will be inaccurate. The detail of processing chest sensor data is presented in the following.

**Figure 7:**
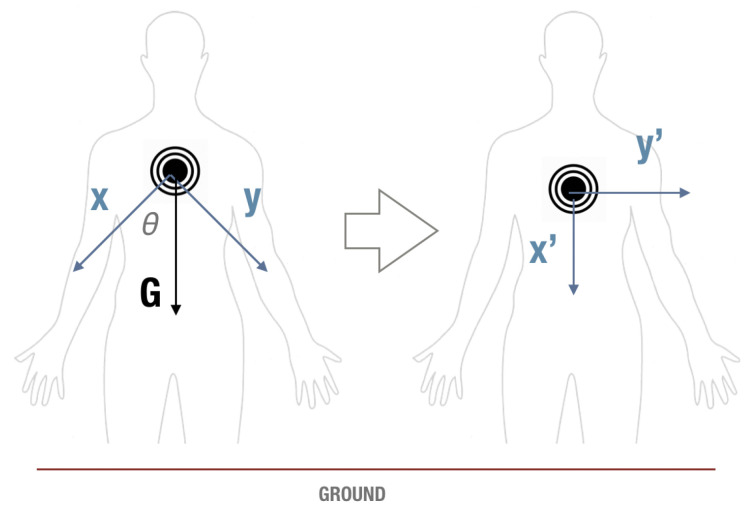
The effect of the rotation matrix. The sensor data will be calibrated into a new X axis which is perpendicular to the ground and a new Y axis which is parallel to the ground.

- Step 1: Calibration
  The shape of our sensor is that of a squat cylinder, like a button, which makes it straightforward to know the flat side should be attached to the chest. However, the orientation of the sensor is not regulated because we design a calibration procedure. The system will first collect the raw inertial data to calibrate the axes of the sensor. The care recipient is requested to stand or sit straight to identify the angles of the chest sensor. A little tilt of the body might affect the calibration. A rotation matrix will be calculated according to Equations (1) and (2) for transforming the data into a base which the X axis is perpendicular to the ground and the Y axis is parallel to the ground. After that, all the data from the chest sensor will be rotated based on the rotation matrix. The effect of the calibration step is shown in Figure 7.
  (1) θ=arctan(y,x)
  (2) $$\left(\begin{array}{c} x^{\prime }\\ y^{\prime } \end{array}\right)\;=\;\left(\begin{array}{cc} \cos\theta & \sin\theta \\ -\sin\theta & \cos\theta \end{array}\right)\left(\begin{array}{c} x\\ y \end{array}\right)$$
- Step 2.1: Noise Removal
  The sensors we adopted are still in their developing stage and not as stable as the commercial products. Hence, they returned measurements containing extremely small values sometimes, which could jeopardize the feature extraction results and needs to be removed in advance. The noise will be removed by the following cleaning process in the system. The process calculates the variance of data within a sliding window, and then set it as a threshold. The data that contain values less than the threshold are removed for noise cleaning.
- Step 2.2: Feature extraction
  The system calculates the average value of the data within the sliding window to obtain features of each axis. The sliding window approach smooths the data, which implicitly applies a second noise removal on the data. The window size is set to one second and no overlapping between windows.
- Step 3: Differentiate Standing or Lying Position
  The dominating axis is decided by comparing the magnitude of the feature of each axis. If Y’ or Z is the dominating axis, it indicates that a care recipient is in the lying position.
- Step 4: Recognize Sleep Posture
  Sleep postures are estimated based on the positive or negative of Y${}^{\prime }$ axis and Z axis, as shown in Table 2.

### 4.2. Wrist Sensor

The characteristics of the wrist motion are obtained by performing feature extraction on the wrist sensor data. The time series data are first segmented into chunks by a sliding window approach, and then each chunk is averaged to obtain the mean value. The values are so-called feature data of wrist position in the rest of this paper.

The collected raw data from wrist sensor are recorded in the form of {timestamp,value1}{value2,value3}, where the timestamp is in milliseconds and the value is floating point. The three values represent the detected acceleration in three axes, which are X, Y and Z axis. We denote these values with $x_{t,j}$, where *t* is the timestamps and j∈{x,y,z} denotes the axis. We let $x_{t}=\left\{x_{t,x},x_{t,y},x_{t,z}\right\}$ to denote a raw data record. The refore, the entire raw data D can be represented as:

(3) $$D=\{x_{t_{1}},x_{t_{2}},x_{t_{3}}...x_{T}\},$$

where *T* denotes the last timestamp in the whole dataset D. The n, we group the raw data into $N=\left\lfloor T/windowsize\right\rfloor$ frames, a frame of data is denoted by

(4) $$f^{i}=\left\{x_{t}\right\},windowsize\times \left(i-1\right)\leq t<windowsize\times i,$$

where $i\in \left[1,N\right]$ is the frame index. In other words, a frame is a collection of raw data in the time duration-windowsize, and the frames are parts of the whole dataset. The windowsize was set to 1000 in our system.

After segmenting the raw data into blocks, we need to obtain the features of each block, namely, to obtain the characteristic of wrist movement, and learn the model for predicting sleep postures. We denote the mean of the *j*-th axis of *i*-th frame as $\mu_{j}^{i}$, where

(5) $$\mu_{j}^{i}=\frac{1}{n_{i}}\sum x_{t,j},\;\forall x_{t,j}\in f^{i}.$$

The $n_{i}$ represents the number of data points in the corresponding sliding window. The feature set *S* for learning the model is $\left\{\mu_{x},\;\mu_{y},\;\mu_{z}\right\}$, which are the means of each axis. We denote the feature set as $X^{i}=\left\{\mu_{x}^{i},\;\mu_{y}^{i},\;\mu_{z}^{i}\right\}$, which is the combination of feature from each axis. Then, we form the feature data matrix X by concatenating all the frame features. Thus, the feature data is present as:

(6) $$X=\left[\begin{array}{c} X^{1}\\ X^{2}\\ \vdots \\ X^{N} \end{array}\right].$$

Let $Y^{i}$ be the posture label of the feature data $X^{i}$, where $Y^{i}\in \{Stand,Supine,Right\;Lateral,$LeftLateral,Prone}. The sleep posture labels are referred to as the *target labels* or *classes*. The labels can form a label column:

(7) $$Y=\left[\begin{array}{c} Y^{1}\\ Y^{2}\\ \vdots \\ Y^{N} \end{array}\right].$$

Finally, the feature data and the corresponding target labels are obtained for model learning.

## 5. Learning Algorithms

**Figure 8:**
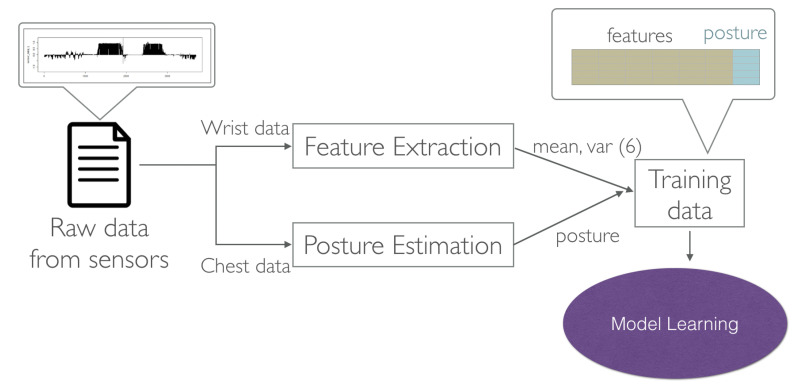
Model training process in the system. The training data and the label data are collected by the smartphone, and then sent to the server for training personal model for posture prediction.

In the last section, the proposed data processing framework transforms the raw data into structural training data. The target labels of sleep postures are obtained from the chest sensor data. The feature data are extracted from the wrist sensor data. The target labels and the feature data can be combined, and a designed matrix is formed for training a classifier using standard learning algorithms. Figure 8 shows the training process of sleep postures. In this paper, two classification algorithms are tested for posture monitoring: Support Vector Machine and Random Forest. Both algorithms will be briefly introduced in this section, and then evaluated in the experiment section.

### 5.1. Random Forest (RF)

The random forest algorithm is adopted for training the prediction model in the proposed system [32]. Random forest is an ensemble learning method that consists of multiple decision tree classifiers. Each decision tree is trained by a randomly selected subset of the training data, which equals the random sampling method with replacement. When constructing the decision tree in RF, the split criterion is decided based on a randomly selected feature set *S*. The randomness in the training process can decrease the variance of an RF model. When it comes to the monitoring phase, the features are fed to each decision tree in the forest, and each tree will return a predictive result. The result which is returned by a majority of decision trees represents the predictive result of the entire forest.

The selection of training sets in RF is random, so the accuracy of the trained model varies. In our experiment, we test the RF model multiple times to obtain the range of the accuracy.

### 5.2. Support Vector Machine (SVM)

SVM is a classifier that classifies data by separating two classes with a hyperplane. The classifier tends to maximize the margin between different classes. The feature space of the data will be mapped into a higher dimension so that the data could be linearly separated. In both training and testing phase, a similarity function is needed for determining the similarity between data. The similarity function can be implemented by kernel functions. The kernel function should be selected depending on the insight of data.

Because SVM is a binary classifier, it can only classify two classes. However, the multiple classes classification of SVM can be achieved by the one-against-one (OAO) and one-against-all (OAA) approaches [33]. We adopt the OAA for model training. The OAA method will build $\left|Y^{i}\right|$ SVM models. The training process considers the feature data as the first class if they belong to the $Y^{h}-th$ class. The new class label for the i-th feature data is denoted by $\hat{Y^{i}}$. The considered method is defined as

(8) $$\hat{Y^{i}}=\left\{\begin{array}{cc} 1, & if\;Y^{i}=Y^{h}\\ -1, & otherwise \end{array}\right.$$

The main idea for training a linear SVM is finding a hyperplane which can create the largest margin between two classes. We first set the normal vector of the hyperplane as W and the offset of the hyperplane as *b*. Thus, the constrains for each classes are formulated as follows:

(9) $$\begin{array}{ccc} W\cdot X^{i}+b & \geq & +1-\xi_{i}\;\forall :\hat{Y^{i}}=+1\\ W\cdot X^{i}+b & \leq & -1+\xi_{i}\;\forall :\hat{Y^{i}}=-1\\ & & \xi_{i}\geq 0\;\forall i. \end{array}$$

The formula enforce the hyperplane to separate the two classes and to have at least 1 unit margin to the data. The term $\xi_{i}$ relaxes the margin constrain, which allows a few data to have shorter distance to the hyperplane. We can find the hyperplane that maximize the margin of two classes and separate out most of the data by solving the Lagrangian dual problem

(10) $$\begin{array}{ccc} & & {\mathrm{SVM}}^{\mathrm{OAA}}:\\ & & \;\max_{\lambda }\;L_{D}=\sum_{i=1}^{N}\lambda_{i}-\frac{1}{2}\sum_{i=1}^{N}\sum_{j=1}^{N}\lambda_{i}\lambda_{j}\hat{Y^{i}}\hat{Y^{j}}K\left(X^{i},X^{j}\right)\\ & & \;\sum_{i=1}^{N}\lambda_{i}\hat{Y^{i}}=0\\ & & \;0\leq \lambda_{i}\leq C,\forall i \end{array}$$

where $\lambda =\left\{\begin{array}{cccc} \lambda_{1} & \lambda_{2} & \cdots & \lambda_{N} \end{array}\right\}$ and *C* is a penalty constant. $K(X^{i},X^{j})$ is a kernel function. We use the radial basis function (RBF) as the kernel in our system

(11) $$K\left(X^{i},X^{j}\right)=e^{-\gamma {∥\begin{array}{c} X^{i}-X^{j} \end{array}∥}_{2}^{2}},\;\gamma \geq 0$$

where γ is a parameter in RBF kernel function. The optimization problem in Equation (10) can be solved by sequential minimal optimization [34]. Finally, the remaining work is to establish the decision rules for classifying multiple class data. In an OAA-SVM model which is built for class $Y^{h}$, the predictive result is decided by the sign of the output of formula. For instance, if we have non-labeled data *Z*, the classification process is shown in Equation (12).

(12) $$\begin{array}{c} C\left(Z\right)=sign\{\left(\sum_{i=1}^{N}\lambda_{i}y_{i}K\left(X^{i},Z\right)\right)+b\}\\ class\left(Z\right)=\left\{\begin{array}{cc} Y^{h} & ,\;if\;C(Z)=“+”\\ other & ,\;if\;C(Z)=“-” \end{array}\right. \end{array}$$

However, there are $\left|Y^{i}\right|$ SVM models built in the system, and each model is only responsible for classifying its own class. The refore, the classifying result will be made by the voting results of each model. A vector of size $\left|Y^{i}\right|$ is constructed and initialized for counting votes. The test data *Z* are the input of every model. If the result from the $Y^{i}$ model is $Y^{i}$ itself, the vote for class $Y^{i}$ will be increased by one; otherwise, the votes for other classes will be increased by one. After the voting is done, the class with the majority of votes will be the output result.

## 6. Experimental Settings and Results

### 6.1. Experiment Settings

**Figure 9:**
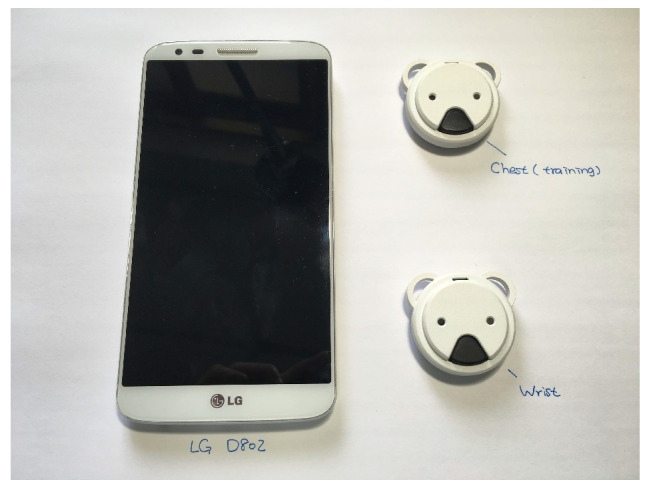
Required hardware in the sleep posture monitoring system. On the left is an Android smartphone, on the right are two wearable sensors.

The proposed system was implemented with Koala sensors and an Android phone, which are shown in Figure 9. The server was implemented by Python, and the package Scikit-learn [35]. The datasets used for evaluating the system are introduced in the following:

- Training Dataset (Labeled Data 1): The dataset is collected with the subjects wearing the chest and wrist sensors, and performing the following two tasks:
  − Sitting on the bed for 3 min, and then lying with the designated four sleep postures.
  − Changing between two sleep postures 10 times.
- Testing Dataset (Labeled Data 2): The testing dataset is collected in an independent two minutes trial. All postures are done by the subject. The ground truth is recorded during the trail. In other words, both sensors are worn during the collecting phase of testing data.

### 6.2. Results

By comparing the estimated postures of the model and the ground truth recorded from the chest data, the results were obtained and reported with confusion matrix (CM). The overall accuracy was calculated by $\frac{\#correctly\;classified\;trials}{\#total\;trials}$, where each trial is a feature vector from a sliding window. Multiple classification models are selected and tested. We discuss the key observations in the following section.

#### 6.2.1. Sleep Posture Monitoring with SVM

**Figure 10:**
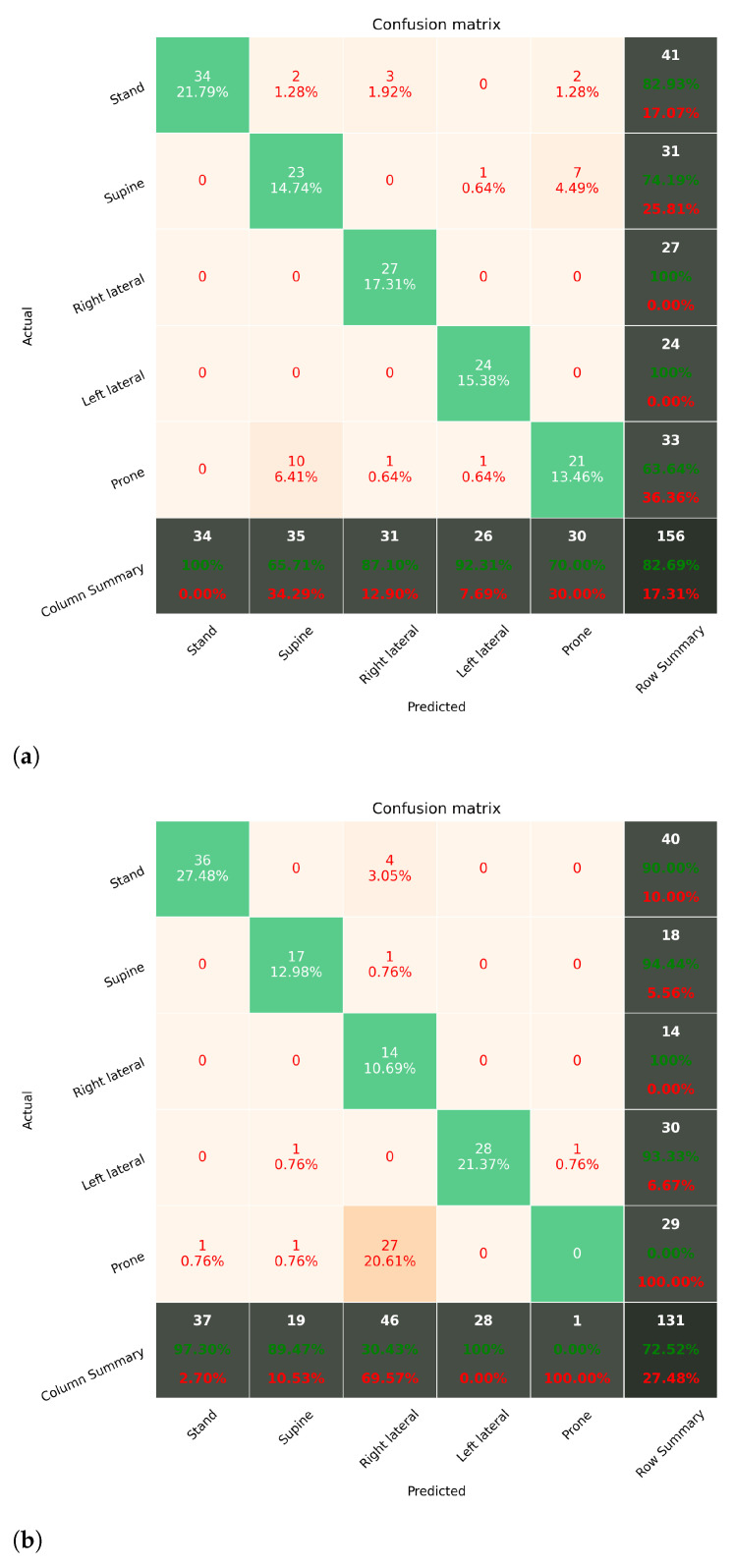
The prediction accuracy of adopting SVC as the learning algorithm. (**a**,**b**) show the confusion matrices of the prediction results of S1 and S2. The overall accuracy of the subjects are 0.82 and 0.72, respectively. (**a**) SVC with subject 1; (**b**) SVC with subject 2.

Figure 10 shows the estimation results of using SVM as the learning algorithm. The results of each subject are presented by CM, where the X-axis presents the true labels, and the Y-axis presents the estimated labels. The values inside the boxes show the number and the proportion of instances which were estimated by the system. The CMs show that most of the instances are classified on the diagonal line, where the correctly estimated instances belong. Among all classes, the “Prone” posture has the lowest accuracy in all subjects. Overall, the estimation accuracies are 0.82 and 0.72 for the subjects, respectively.

#### 6.2.2. Sleep Posture Monitoring with RF

**Figure 11:**
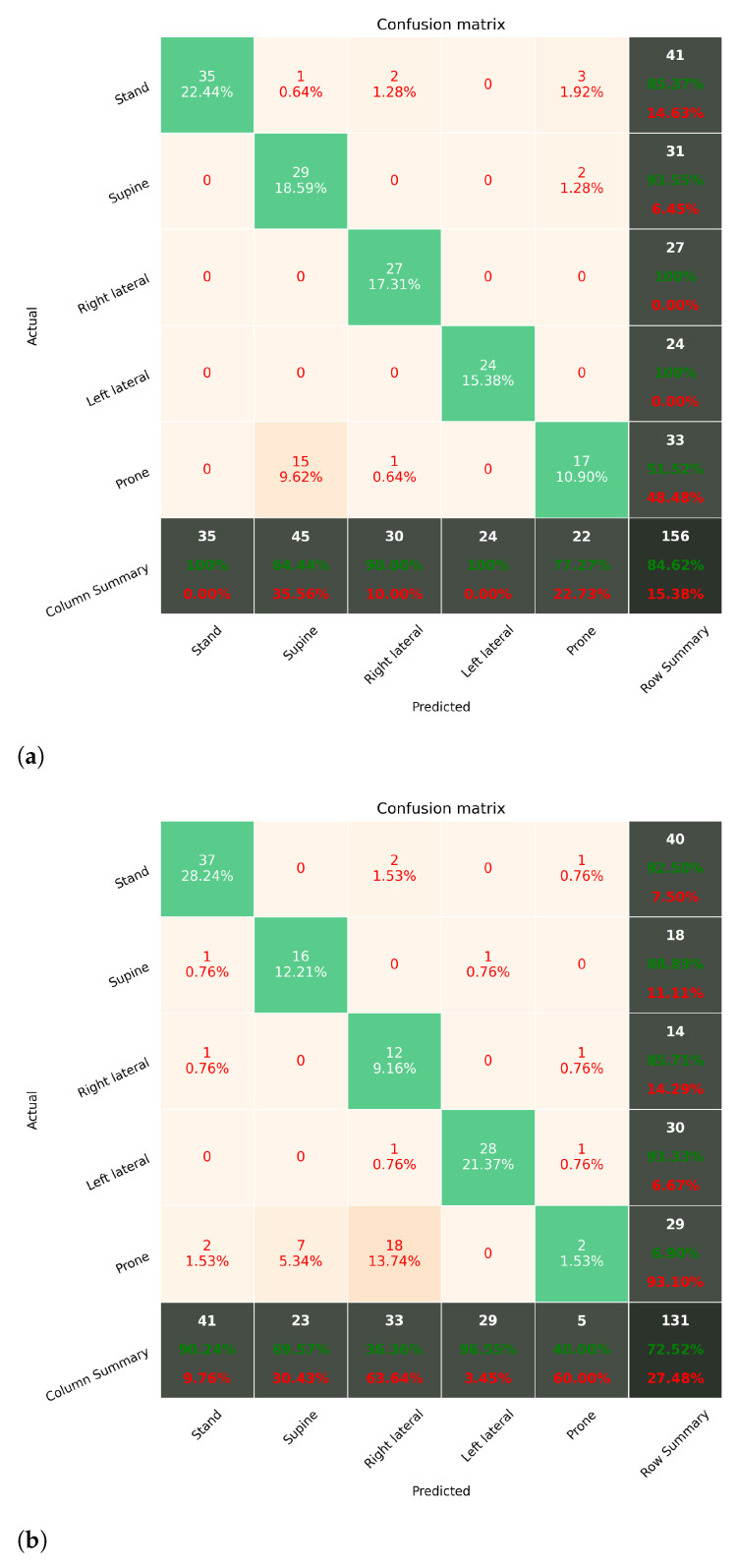
The prediction accuracy of adopting RF as the learning algorithm. (**a**,**b**) show the confusion matrices of the prediction results of S1 and S2. The overall accuracy of the subjects are 0.84 and 0.72, respectively. (**a**) RF with subject 1; (**b**) RF with subject 2.

Figure 11 shows the estimation results of the proposed system when using RF as the learning algorithm. The results are presented in CMs as well. The estimation results show that the accuracy is above 0.85 across all classes except for the prone position, which is similar to the results of using SVM. The overall estimation accuracies are 0.84 and 0.72 for subject 1 and subject 2, respectively.

A parameter of training random forest model is the number of decision trees, which specifies how many decision tree will be constructed in the model. To decide this number, we split the training dataset (Labeled data 1) into two parts: one for training the model with a specific number of trees, and the other for validating the accuracy of this setting. The proportion for splitting the data we adopted is two thirds for training and one third for validation. Our system trains the models with tree numbers from one to fifty, and pick the one with the best accuracy to be the posture classifying model. This procedure can prevent the trained model from overfitting.

**Figure 12:**
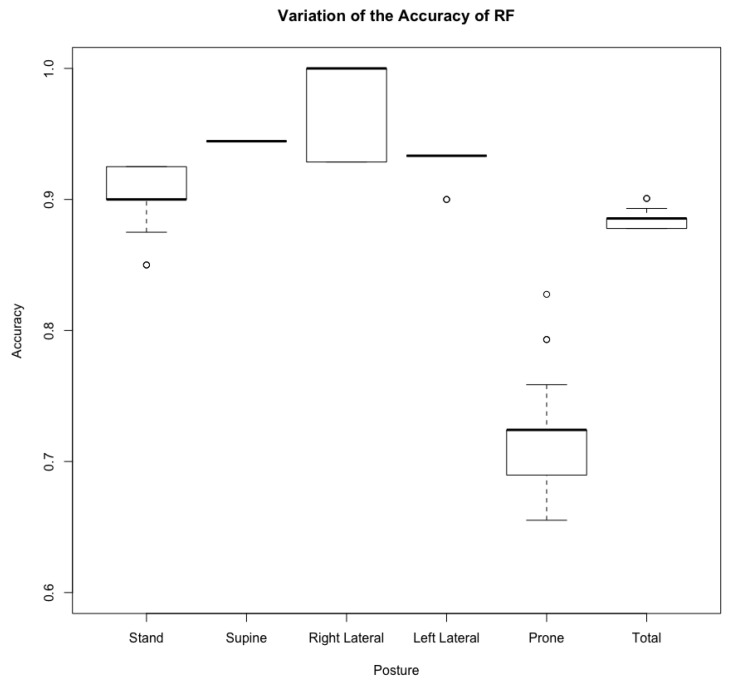
Statistic result for RF model. The boxes represent the 25 percentile and the 75 percentile. The bold horizontal lines represent the mean. The dots represent the outliers.

A further experiment is performed to test how the randomness in RF affects the accuracy. We had done the training and testing phases for a hundred trials, and then analyzed the collected results from each trial. The accuracy for each posture is shown by a box diagram in Figure 12. Note that the overall accuracy of RF varies in one hundred trials.

## 7. Discussion

### 7.1. Generalizability of the System

This section investigates the generalizability of the proposed system on different subjects. An experiment was conducted to evaluate the prediction performance of the proposed system on a new subject. In this experiment, the training phase ran on the existing datasets of other subjects, which is referred to as the source, and the estimation phase was tested on the new subject, which is referred to as the target. Table 3 shows that the largest degradation of accuracy was 0.03, which is not significant. This indicates that the performance was not affected by model transferring across subjects. As a result, the proposed system is able to generalize to other subjects.

### 7.2. Prediction Performance on the Same Subject

Table 4 presents the prediction accuracy of the proposed method on multiple sessions of the same subject. The subject performed the experiment five times for testing data collection. The prediction model was learned from the subject’s training dataset. We can observe that the prediction accuracy was greater than 82% in four out of five sessions, and the average accuracy was 83%. This demonstrates that the proposed method was able to perform accurate sleep posture recognition on the same subject.

### 7.3. Prone Is the Most Confusing Posture

It can be observed that the accuracy for prone posture is significantly lower than other classes in all subjects. In Figure 10 and Figure 11, we can observe that the misclassified trials of prone posture were mainly misclassified to right lateral and supine, depending on subjects. This means the position of the right wrist (where the subject wore the sensor) could be overlapping in both postures, and the model tends to classify them as right lateral (supine) because it has more training data in this situation. To improve the accuracy of estimating the prone posture, a long-term observation has to be performed to obtain the distribution of postures.

### 7.4. Future Applications

Because the proposed system overcomes the disadvantages of the traditional monitoring systems, we can improve many parts of the current monitoring system, or easily been integrated into a ubiquitous healthcare service system [36]. Besides, we list some promising applications in the followings:

#### 7.4.1. Medicine Dosage Adjustment Based on Sleep Posture Profile

Our system can assist the dosage adjustment of medicines that have sleep-related side effects. For example, the overdosing of Parkinson’s disease medicine may make the patient’s body rigid during sleep. In practice, the dosage of the prescribed medicine is often adjusted by considering the severity of patient’s side effect. However, the events that happen during sleep are hard to be perceived by the patients themselves. Our sleep posture monitoring system can record their sleep conditions and assist the physicians in prescribing the correct dosage.

#### 7.4.2. Home Long-Term Sleep Monitoring

Our system is easy to be deployed at home, and the sleep condition is recorded every day. Some of the symptoms may not happen every day. Thus, it takes a long time to identify these symptoms. We can also detect whether the sleep behavior is changed. This might be an indication of sickness. Our system is suitable for these scenarios because of being designed for long-term usage. Large-scale experiments will be further required to investigate the long-term monitoring accuracy.

#### 7.4.3. Risky Position Alarm

An alarm function can be easily integrated with our monitoring system to prevent sleep-related diseases. The duration of sleep postures are presented by our system in real-time. Therefore, the alarm system only needs physicians or caregivers to set the condition when patients need to be alarmed.

## 8. Conclusions

This paper proposed a cost-effective and user-friendly sleep posture monitor system. We designed a system that automatically recognizes the body posture for labeling the data during the training phase, which avoids labor-intensive labeling works. In the monitoring phase, our system can recognize the posture by analyzing data collected from one sensor on the wrist, and perform accurately. We tested two different types of learning algorithms with our system, and achieved over 70% accuracy with random forest algorithm, and 73% accuracy with SVM algorithm, respectively. In addition, the proposed system is more cost-effective than the existing approaches which use a pressure mat, camera or other dedicated equipment. In summary, we demonstrated that the proposed the iSleePost system using only the wrist accelerometer can accurately monitor sleep postures, providing important insights into the design of heath monitoring systems. Many important and interesting research topics can be extended from this study in the future, such as elder healthcare and medicine dosage adjustment based on sleep posture profile.

## Figures and Tables

**Table 1:** A comparison between the proposed method and the related works.

Type		Equipment	Labeling	Year	Paper/Product
Non-contact		Camera	Manual	2017	[19]
		Depth-camera		2014	[20]
		Depth-camera		2016	[21]
		Wi-Fi		2014	[22]
Contact	Non-wearable	Pressure mat		2015	[23]
		Pressure mat		2016	[24]
		Hydraulic sensors		2018	[25]
		Piezoelectric sensor		2018	[26]
		pressure mat and infrared sensors		2018	[27]
	Wearable	Inertial motion sensor		2015	[14]
		Inertial motion sensor		2010	Leaf Healthcare [28]
		Tilt sensor		2008	[15]
		Inertial motion sensor	Automatic	2020	**Proposed**

**Table 2:** Decision manner.

Dominating Axis	+/−	Body Posture
Y’	Positive	Left Lateral
	Negative	Right Lateral
Z	Positive	Supine
	Negative	Prone

**Table 3:** A cross-subject experiment was performed on the subjects to evaluate the generalization of the method. The accuracies decreased 0.03 at most when transferring models across subjects.

		Target	
		Subject 1 Testing	Subject 2 Testing
Source	Subject 1 Training	0.80 (0.03)/0.83	0.83 (0.01)/0.82
	Subject 2 Training	0.70 (0.02)/0.75	0.73 (0.02)/0.73

**Table 4:** The prediction performance of the proposed method on the same subject. Four out of five sessions had a prediction accuracy grater than 82%.

Session Name	Accuracy
S1-1	0.8244
S1-2	0.9464
S1-3	0.8405
S1-4	0.6005
S1-5	0.9571
Avergae	0.8338

## Data Availability

The raw sensory data are available on: https://tinyurl.com/y8kfyv5k.
